# Mortality in functional seizures: Evidence from a large electronic health records dataset

**DOI:** 10.1002/epi.70230

**Published:** 2026-03-31

**Authors:** Richard A. Kanaan, Rok Berlot, Tom Pollak, Markus Reuber, Stoyan Popkirov

**Affiliations:** ^1^ Department of Psychiatry University of Melbourne, Austin Hospital Heidelberg Victoria Australia; ^2^ King's College London Institute of Psychiatry, Psychology, and Neuroscience London UK; ^3^ Department of Neurology University Medical Center Ljubljana Ljubljana Slovenia; ^4^ Faculty of Medicine University of Ljubljana Ljubljana Slovenia; ^5^ Academic Neurology Unit University of Sheffield, Royal Hallamshire Hospital Sheffield UK; ^6^ Department of Neurology and Center for Translational Neuro‐ and Behavioral Sciences University Hospital Essen, University of Duisburg‐Essen Essen Germany

**Keywords:** etiology, functional neurological disorder, mechanism, nosology

## Abstract

**Objective:**

Several studies have found that people with functional seizures (FS) have increased mortality, approaching that of epilepsy (epileptic seizures [ES]). The small numbers of deaths in these studies make it unclear whether they can be attributed to comorbidities. We used a very large electronic health database to compare mortality in FS and ES, controlling for comorbidity.

**Methods:**

We searched the TriNetX database for people with FS and no ES, people with ES and no FS, and people with neither FS nor ES (NS). We compared mortality while controlling for demographics and comorbid conditions.

**Results:**

We identified 1 916 787 people with ES, 32 854 people with FS, and 21 053 667 healthy controls. FS had rates of mental and physical comorbidities that were higher than NS and, for most categories, higher than ES. The risk of death (hazard ratio [HR]) in ES compared to FS was 2.99 (95% confidence interval [CI] = 2.81–3.18), which was reduced to 2.07 (95% CI = 1.92–2.24) when matched for 31 demographic and health factors. The risk of death in NS compared to FS was .56 (95% CI = .53–.59); after matching for demographics and mood disorders, this was .48 (95% CI = .44–.53). Matched sensitivity analyses suggest this was particularly pronounced in the year following diagnosis (HR = .39, 95% CI = .33–.47) and in the 5th decade of life (HR = .38, 95% CI = .27–.52).

**Significance:**

FS confers approximately doubled mortality over that conferred by its comorbidities, although still only half of that conferred by ES, most prominently after diagnosis and in early middle age.

**Key points**

- People with functional seizures have even higher rates of most mental and physical health comorbidities than people with epilepsy.
- People with functional seizures have twice the mortality of people without seizures but only half that of people with epilepsy, even after controlling for comorbidity.
- The increase in mortality in functional seizures is most marked after diagnosis and in the 5th decade of life.

## 1 INTRODUCTION

People with functional seizures (FS) commonly report that their illness is not taken seriously by clinicians.^1^ This appears particularly problematic in light of recent studies suggesting that FS are associated with substantially increased mortality,^2^, ^3^, ^4^, ^5^ with some estimates approaching the premature mortality rates seen in people with epilepsy. However, although these were large studies for the field, the number of deaths in each was relatively small, making it hard to tease apart what might be causing any increase.

One potential contributor to increased mortality in individuals with FS disorders is the high rate of comorbidities, particularly of psychiatric disorders. We increasingly understand that these confer greater mortality, not just from suicide, or the lifestyle risks they implicate, but seemingly from direct effects of the disorders on, for example, the cardiovascular system.^6^ It remains unclear whether the diagnosis of FS confers any additional risk to that conferred by the comorbid conditions from which people with FS are known to suffer.^4^, ^7^, ^8^

We sought to use a much larger clinical database to explore the mortality of FS further. TriNetX is a data repository including the electronic health records of more than 160 million patients from 19 countries of the Americas, Europe, the Middle East, Asia, and Australasia.^9^ In this study, we set out to interrogate the TriNetX database, comparing mortality in people with FS, in those with epilepsy (epileptic seizures [ES]), and in those without a seizure disorder (NS). We hypothesized that FS would be associated with higher rates of comorbid mental disorders than either ES or NS; that FS subjects would suffer increased mortality compared with NS, but that this would be fully accounted for by comorbid illnesses as previously reported^8^; and that the mortality would remain below that of ES.

## 2 MATERIALS AND METHODS

### 2.1 Study population

The TriNetX Research Network is updated live, so that the total number of patients and health care organizations varies over time, depending on which are online. We accessed the database on July 19, 2025, at approximately 17:00 Australian Eastern Standard Time; at that time, TriNetX was reporting data from more than 160 million patients from 152 health care organizations. We searched for individuals with a diagnosis of FS but no diagnoses of ES at any time (International Classification of Diseases, 10th Revision, Clinical Modification [ICD‐10‐CM] codes F44.5 and G40 respectively) and compared them with individuals with a diagnosis of ES and no diagnosis of FS, and with a group of “normal” subjects, without seizure disorders (NS). The NS set was defined as those meeting ICD‐10 code Z00.0 (encounter for a general adult medical examination without abnormal findings), with no diagnosis of ES or FS. Individuals with unknown gender were excluded from all groups, and TriNetX excludes individuals whose index diagnosis occurred 20 years ago or more from analysis.

Defining the groups using ICD‐10 diagnoses is imperfect. The F44.5 code for FS has been shown to be highly specific (96%) in electronic health care records,^10^ but due to concerns over the potential miscoding of FS by error in some US electronic health records,^11^ a sensitivity analysis was conducted on the group defined by those with diagnoses of FS made on at least two occasions,^7^ as the chance of the same accidental coding occurring twice would be much lower. The specificity of epilepsy diagnoses is lower, at 83%, and although it can be increased by algorithms that, for example, add prescription data, the gain is marginal (84%).^12^ The greatest diagnostic risk for each is to be misdiagnosed as the other, so we excluded the group with diagnoses of both FS and ES from all analyses to further improve the reliability of the groupings. This does mean that the additional comparison group of those with ES and FS could not be included. Although it is common for people with ES to have comorbid FS, TriNetX will not reliably differentiate between those with comorbidity and those whose initial diagnosis has been revised, so a group with both diagnoses would represent a mixture of unknown composition.

### 2.2 Measures

We examined ICD‐10‐CM main categories (designated by letters) for physical comorbidities and subcategory root codes for mental and behavioral disorders (F category); as FS are part of the F40–49 subcategory, individual codes from that subcategory were used (see Table 1). ICD‐9‐CM codes are mapped to ICD‐10‐CM codes using the General Equivalence Mappings from the Centers for Medicare & Medicaid Services. Comorbid conditions were captured if diagnosed in the 3 years up to and including the day of the index diagnosis (or, for the NS group, the day of the medical examination). As almost every category of comorbidity has been found to be elevated in FS, we used the full range of categories in ICD‐10. We used Deceased as the outcome, measured at any time after the index diagnosis, with a sensitivity analysis with outcomes limited to those occurring within 1 year of diagnosis.

**TABLE 1:** Demographics and clinical conditions present in people with ES (*n* = 1 916 787) and FS (*n* = 32 854).

Demographics						
Cohort	Characteristic	Mean ± SD	Patients	% of cohort	*p*	Std diff
ES FS	Age at index	40.4 ± 25.2 34.7 ± 19.5	1 809 234 32 724	100% 100%	<.001	.252
ES FS	Male		915 989 8147	50.6% 24.9%	<.001	.551
ES FS	White		1 025 832 19 147	56.7% 58.5%	<.001	.037
ES FS	Black or African American		268 322 4756	14.8% 14.5%	.134	.008
ES FS	Asian		60 004 446	3.3% 1.4%	<.001	.130

Comorbid conditions						
Cohort		ICD‐10 code and category	Patients	% of cohort	*p*	Std diff
ES FS	F30–F39	Mood (affective) disorders	179 539 8371	9.9% 25.6%	<.001	.419
ES FS	F10–F19	Mental and behavioral disorders due to psychoactive substance use	181 270 4297	10.0% 13.1%	<.001	.097
ES FS	F50–F59	Behavioral syndromes associated with physiological disturbances and physical factors	21 811 1442	1.2% 4.4%	<.001	.195
ES FS	F90–F98	Behavioral and emotional disorders with onset usually occurring in childhood and adolescence	42 673 2478	2.4% 7.6%	<.001	.242
ES FS	F80–F89	Pervasive and specific developmental disorders	57 883 976	3.2% 3.0%	.027	.013
ES FS	F20–F29	Schizophrenia, schizotypal, delusional, and other nonmood psychotic disorders	36 316 1065	2.0% 3.3%	<.001	.078
ES FS	F60–F69	Disorders of adult personality and behavior	17 708 1247	1.0% 3.8%	<.001	.186
ES FS	F70–F79	Intellectual disabilities	18 988 235	1.0% .7%	<.001	.035
ES FS	F40	Phobic anxiety disorders	5840 438	.3% 1.3%	<.001	.112
ES FS	F41	Other anxiety disorders	145 072 8635	8.0% 26.4%	<.001	.502
ES FS	F43	Reaction to severe stress, and adjustment disorders	44 778 3584	2.5% 11.0%	<.001	.344
ES FS	F42	Obsessive–compulsive disorder	5016 420	.3% 1.3%	<.001	.115
ES FS	F45	Somatoform disorders	4820 600	.3% 1.8%	<.001	.154
ES FS	J00–J99	Diseases of the respiratory system	384 930 8774	21.3% 26.8%	<.001	.130
ES FS	M00–M99	Diseases of the musculoskeletal system and connective tissue	421 758 11 248	23.3% 34.4%	<.001	.246
ES FS	E00–E89	Endocrine, nutritional, and metabolic diseases	447 830 9349	24.8% 28.6%	<.001	.086
ES FS	I00–I99	Diseases of the circulatory system	458 602 7977	25.3% 24.4%	<.001	.022
ES FS	K00–K95	Diseases of the digestive system	376 075 8912	20.8% 27.2%	<.001	.151
ES FS	N00–N99	Diseases of the genitourinary system	321 623 7397	17.8% 22.6%	<.001	.120
ES FS	G00–G99	Diseases of the nervous system	444 387 12 109	24.6% 37.0%	<.001	.272
ES FS	A00–B99	Certain infectious and parasitic diseases	236 846 5188	13.1% 15.9%	<.001	.079
ES FS	L00–L99	Diseases of the skin and subcutaneous tissue	207 577 4906	11.5% 15.0%	<.001	.104
ES FS	H00–H59	Diseases of the eye and adnexa	173 908 3871	9.6% 11.8%	<.001	.072
ES FS	D50–D89	Diseases of the blood and blood‐forming organs and certain disorders involving the immune mechanism	214 865 3674	11.9% 11.2%	<.001	.020
ES FS	H60–H95	Diseases of the ear and mastoid process	118 875 2782	6.6% 8.5%	<.001	.073
ES FS	O00–O9A	Pregnancy, childbirth, and the puerperium	25 589 1121	1.4% 3.4%	<.001	.131

Abbreviations: ES, epilepsy; FS, functional seizures; ICD‐10, International Classification of Diseases, 10th Revision; Std Diff, standardized difference.

### 2.3 Statistical analysis

We report the total numbers and proportions of patients from each cohort who had the corresponding diagnostic code(s). Comparisons between cohorts used chi‐squared tests, independent samples *t*‐tests, and *Z*‐tests. Where indicated, the cohorts were matched one‐to‐one on demographic variables using propensity scores from the specified covariates. Outcome analyses are presented as both risk ratios (the ratio of the two risks of death over the whole study period) and hazard ratios (the ratio of the two risks at a given moment). Analyses were conducted using the TriNetX statistical engine, which imposes some restrictions on the analysis of relatively small or very large datasets. Notably, the number of covariates used for matching is limited by the total number of cases in the cohorts; because of the extremely large size of the healthy control dataset (more than 20 million individuals), propensity score matching was not possible on the full set of covariates. Propensity score matching was therefore conducted on the full dataset but on a limited set of variables, and full matching conducted on the dataset divided up into decades (10–19 years old, 20–29 years old, etc.).

### 2.4 Ethics

The TriNetX data are publicly available and deidentified according to the standard in Section §164.514(b) (1) of the Health Insurance Portability and Accountability Act Privacy Rule (http://trinetx.com) for retrospective analysis. The database does not allow access to individual records but provides aggregate reports and statistical analyses from the database's own statistical engine. The study was approved by the Austin Health Research Ethics Committee (HREC/28971/Austin‐2025).

## 3 RESULTS

### 3.1 People with ES compared with people with FS

People with FS showed significantly higher rates of all prediagnosis mental health disorders than those with ES, other than intellectual disabilities and developmental disorders, and all categories of physical health disorder other than diseases of the circulatory system or the blood.

Despite the higher rates of most comorbidities in FS, the relative risk of death occurring at any point after diagnosis in ES compared with FS was 3.86 (95% confidence interval [CI] = 3.635–4.098), encompassing 217 193 deaths in ES and 1040 deaths in FS, with a hazard ratio of 2.99 (95% CI = 2.810–3.175). Propensity score matching the cohorts on all the demographic characteristics and comorbid conditions in Table 1 yielded matched cohorts of 32 711 each. Repeating the mortality analysis on these matched cohorts gave a reduced relative risk of death in ES compared with FS of 2.96 (95% CI = 2.748–3.179), representing 2740 deaths in ES and 927 in FS, with a reduced hazard ratio of 2.07 (95% CI = 1.922–2.235).

### 3.2 NS compared with people with FS

People with FS showed significantly higher rates of all comorbid mental and physical health disorders than NS (Table 2).

**TABLE 2:** Demographics and clinical conditions present in those without seizure disorders (*n* = 21 053 677) and those with FS (*n* = 32 854).

Demographics						
Cohort	Characteristic	Mean ± SD	Patients	% of cohort	*p*	Std diff
NS FS	Age at index	35.2 ± 25.0 34.7 ± 19.5	20 526 738 32 724	100% 100%	<.001	.022
NS FS	Male		9 363 535 8147	45.6% 24.9%	<.001	.444
NS FS	White		11 576 999 19 147	56.4% 58.5%	<.001	.043
NS FS	Black or African American		2 542 563 4756	12.4% 14.5%	<.001	.063
NS FS	Asian		979 690 446	4.8% 1.4%	<.001	.199

Diagnosis						
Cohort		ICD‐10 code and category	Patients	% of cohort	*p*	Std diff
NS FS	F30–F39	Mood (affective) disorders	1 131 953 8371	5.5% 25.6%	<.001	.576
NS FS	F10–F19	Mental and behavioral disorders due to psychoactive substance use	831 200 4297	4.0% 13.1%	<.001	.328
NS FS	F50–F59	Behavioral syndromes associated with physiological disturbances and physical factors	183 560 1442	.9% 4.4%	<.001	.220
NS FS	F90–F98	Behavioral and emotional disorders with onset usually occurring in childhood and adolescence	319 543 2478	1.6% 7.6%	<.001	.291
NS FS	F80–F89	Pervasive and specific developmental disorders	121 513 976	.6% 3.0%	<.001	.181
NS FS	F20–F29	Schizophrenia, schizotypal, delusional, and other nonmood psychotic disorders	88 588 1065	.4% 3.3%	<.001	.211
NS FS	F60–F69	Disorders of adult personality and behavior	58 075 1247	.3% 3.8%	<.001	.251
NS FS	F70–F79	Intellectual disabilities	18 978 235	.1% .7%	<.001	.099
NS FS	F41	Other anxiety disorders	1 183 951 8635	5.8% 26.4%	<.001	.585
NS FS	F43	Reaction to severe stress, and adjustment disorders	275 442 3584	1.3% 11.0%	<.001	.408
NS FS	F42	Obsessive–compulsive disorder	29 837 420	.1% 1.3%	<.001	.135
NS FS	F45	Somatoform disorders	29 531 600	.1% 1.8%	<.001	.171
NS FS	J00–J99	Diseases of the respiratory system	4 213 691 8774	20.5% 26.8%	<.001	.148
NS FS	M00–M99	Diseases of the musculoskeletal system and connective tissue	4 261 249 11 248	20.8% 34.4%	<.001	.308
NS FS	E00–E89	Endocrine, nutritional, and metabolic diseases	4 090 645 9349	19.9% 28.6%	<.001	.203
NS FS	I00–I99	Diseases of the circulatory system	3 527 873 7977	17.2% 24.4%	<.001	.178
NS FS	K00–K95	Diseases of the digestive system	3 271 668 8912	15.9% 27.2%	<.001	.277
NS FS	N00–N99	Diseases of the genitourinary system	3 104 845 7397	15.1% 22.6%	<.001	.192
NS FS	G00–G99	Diseases of the nervous system	2 581 784 12 109	12.6% 37.0%	<.001	.590
NS FS	A00–B99	Certain infectious and parasitic diseases	2 138 330 5188	10.4% 15.9%	<.001	.161
NS FS	L00–L99	Diseases of the skin and subcutaneous tissue	2 390 423 4906	11.6% 15.0%	<.001	.099
NS FS	H00–H59	Diseases of the eye and adnexa	1 421 655 3871	6.9% 11.8%	<.001	.169
NS FS	D50–D89	Diseases of the blood and blood‐forming organs and certain disorders involving the immune mechanism	1 190 451 3674	5.8% 11.2%	<.001	.195
NS FS	H60–H95	Diseases of the ear and mastoid process	1 358 834 2782	6.6% 8.5%	<.001	.071
NS FS	O00–O9A	Pregnancy, childbirth, and the puerperium	370 173 1121	1.8% 3.4%	<.001	.102

Abbreviations: FS, functional seizures; ICD‐10, International Classification of Diseases, 10th Revision; NS, no seizure disorder; Std Diff, standardized difference.

There was no significant difference in risk of death between groups (risk ratio = 1.015, 95% CI = .956–1.078), representing 627 756 deaths in NS and 1040 in FS, although the hazard ratio was .56 (95% CI = .526–.594). Due to the size of the NS group, propensity score matching was limited to four characteristics: age, gender, White race, and mood disorders, yielding matched cohorts of 32 274 each. After matching, the risk ratio was .83 (95% CI = .756–.914), representing 770 deaths in NS and 927 in FS, and the hazard ratio again lower at .48 (95% CI = .438–.533).

### 3.3 Sensitivity analysis of 1‐year outcomes

This discrepancy between risk and hazard ratios in the above suggests that, although similar numbers of people are dying in the two groups, death is occurring sooner in people with functional seizures. This is supported by the time in follow‐up, which differed markedly between healthy controls (median = 1163 days) and people with functional seizures (516 days), and suggests that similar numbers of deaths are being observed in the patient group in half the time. Restricting the follow‐up period to 1 year tended to confirm this suspicion: with the median follow‐up period identical, at 365 days, the risk ratio was less than half (.45, 95% CI = .377–.544), much closer to the hazard ratio of .39 (95% CI = .326–.472).

### 3.4 NS compared with people with FS by decade

Risk ratio of deaths for those aged in each decade are shown below, both raw, and matched on the same 31 characteristics as in ES versus FS analysis, showing substantially lower risks and rates of death in NS than in people with FS, particularly in the 5th decade, although not all were significant (Table 3).

**TABLE 3:** Risk and hazard ratios, plus 95% confidence intervals, for those without seizures compared with people with functional seizures, by decade.

Decade	Unmatched risk ratio	Matched risk ratio	Matched hazard ratio
10–19	.419 (.256–.684)	.813 (.391–1.687)	.567 (.271–1.186)
20–29	.506 (.374–.686)	.805 (.509–1.272)	.598 (.376–.949)
30–39	.357 (.285–.448)	.824 (.588–1.155)	.627 (.446–.882)
40–49	.314 (.263–.376)	.486 (.352–.672)	.379 (.274–.525)
50–59	.450 (.387–.525)	.728 (.570–.930)	.553 (.430–.710)
60–69	.564 (.494–.645)	.809 (.655–.998)	.585 (.470–.727)

### 3.5 Sensitivity analysis of those with two diagnoses of FS

The group receiving two FS diagnoses were approximately one third of the whole FS group (12 189 people). They were younger, more likely to be White and female, and had higher rates of comorbid diagnoses in all categories of illness (see Table S1). Healthy controls had a higher risk of death (risk ratio = 1.25, 95% CI = 1.119–1.401) than those with two diagnoses of FS; their hazard ratio was .79, however (95% CI = .708–.889). After matching for age, gender, White race, and mood disorders, the risk ratio declined to .798, (95% CI = .670–.950) and hazard ratio to .476 (95% CI = .396–.572).

## 4 DISCUSSION

The risk of death was much higher in epilepsy than in FS, even though FS subjects had higher rates of most comorbid mental and physical illnesses. The risk of death in FS and in NS was closer, although people with FS had significantly higher rates of all categories of mental and physical illness than healthy controls. After matching for demographic variables and mood disorders, healthy control subjects were only at slightly lower risk of death than people with FS. Although, as people with FS were followed up for a shorter period of time, on average, it is likely that these deaths occurred more quickly—twice as fast as in healthy controls. Examining the risk by decade, it appeared that the greatest disparity between the FS and NS groups arose in the 5th decade, where the risk of death in those with a diagnosis of FS was more than double that of healthy controls, even when controlling for all categories of physical or mental comorbidity. Restricting the group of people with FS to those who had received the diagnosis on at least two separate occasions did not greatly alter the ratios.

There is a disparity between the risk ratio and hazard ratio in most of these analyses. These appear to be due to differences in how long subjects were followed up in the TriNetX system. Although we defined our outcomes as occurring at any point, our “healthy control” subjects were “observed” by TriNetX for longer periods of time, and thus had more chance of a death being captured, so that the hazard ratio is perhaps the more useful measure of the two. By that measure, the risk of death in epilepsy was twice that of FS, which was twice that of those without any seizure disorder. Causes of death are not available within the TriNetX database, and the reasons for these differences therefore remain unclear, but the differences remained even after controlling for mental and physical health comorbidities. Our second hypothesis was therefore not supported; FS appears to confer excess mortality risk in addition to that of its many comorbidities. As the effect is particularly strong in the year following diagnosis, this may suggest it commonly reflects a response to the diagnosis, or perhaps to the factors that led to the onset of FS.

The extent of the comorbidities in FS was nonetheless surprising. Although psychiatric comorbidity is commonly reported,^13^ the range and extent of psychiatric comorbidities was striking; and although the physical comorbidities have been reported previously,^4^ our estimates appear larger, even larger than in ES in most cases. They were, moreover, in our study, already present at the time of diagnosis, which makes them less likely to be consequences of the disorder. It is tempting to see this as the encoding of somatoform diagnoses in physical categories, rather than the mental category (where somatoform comorbidities were relatively rare, at only 1.8%), but it demands an explanation that would apply across the board, to infections or disorders of the eye as much as to the musculoskeletal or digestive systems.

It is unclear why people with FS should be followed up for so much shorter time than other people. Although the excess death may account for some of the discrepancy, death remains rare, even in this group, and can only account for a small fraction. It suggests that, for whatever reason, people with FS have less sustained engagement with the health care providers of the TriNetX network after diagnosis. A degree of social instability is a feature of many mental health disorders, but the difference in follow‐up was present even when matching for mental health comorbidity. This may be further evidence of the particular dissatisfaction that many people with FS have with medical services,^1^ disengaging perhaps because they have been given a diagnosis they find unwelcome, or because of how it is subsequently managed.^14^ Alternatively, it may reflect that services for FS are often rudimentary compared with ES, and that patients are likely to be discharged from the (epilepsy) service that diagnosed them without ever engaging in appropriate follow‐up.^15^ Why our NS group should also have more extensive engagement than FS is more speculative, although it is perhaps likely that people who attend for medical examinations where no abnormalities are detected are those who are particularly help‐seeking, or particularly well engaged with their health care providers.

It is intriguing that the 5th decade should present particular risk for people with FS, once matched for comorbidities. Other studies have found suicide to be a particular risk in those with FS younger than 50 years,^3^ although why it should be more of a problem in the 40s than the 30s is open to speculation; it is of course the time of the “midlife crisis,” and when women, in particular (who of course make up the majority of people with FS, although not necessarily of its deaths), may have to adapt to changes in social role with menopause, or a return to the workforce after motherhood, but these have not previously been identified as challenges particularly affecting FS. Suicide is a common cause of early death in FS,^5^ and the 5th decade is the peak for suicide in women in general for many reasons^16^; the many comorbidities identified in this study may imply a cumulative effect in FS, with comorbidities and adversities combining. The sheer number of comorbidities identified in FS makes multimorbidity^17^ highly likely, with effects on mortality that may prove far more than the sum of their individual risks.^18^

The most challenging question was the choice of a “healthy control” dataset. As a database of clinical encounters, TriNetX is a dataset of ill health, almost by definition, and TriNetX does not allow groups to be defined solely by an absence (of a seizure disorder, in our case). There are ways around this, such as the methods we employed, but they all involve challenges. The dataset we used was a group of subjects who consulted a doctor and were found to have no health complaints. They are perhaps therefore a particularly healthy group, and any mortality differences may be inflated thereby. Other options included the group of people who were selected to be “healthy controls” in clinical trials, or people who have a health complaint that might be thought unlikely to substantially impact mortality, such as an ingrown toenail or a bunion. It was clear when exploring these, however, that although they gave broadly similar outcomes, the vast size of the TriNetX database meant that substantial differences were detectable in comorbidities, and any difference in outcomes between these would likely be of significance. Although the size of our control group paradoxically limited the amount of matching we could perform, it was felt to be the least biased. The rates of major mental health disorders (anxiety, affective disorders, schizophrenia) found in our NS sample are roughly equal to global prevalence rates reported elsewhere,^19^ suggesting that at least in that regard our NS sample corresponds to the general population.

There are many limitations to a study addressing mortality in this way. Mortality data within TriNetX are incomplete, as deaths occurring outside of participating health care organizations are unlikely to be captured. Additionally, data are limited to all‐cause mortality, with no reliable information on specific cause of death. Missing from the database (at least, not made available to us) are socioeconomic measures, which are likely to confound many of the associations, as well as predict mortality independently.^20^ Finally, differential follow‐up and site heterogeneity may bias estimates. There are also significant limitations to the quality of the diagnoses in a clinical database. The likelihood of miscoding of all seizures as FS has already been noted^11^; conversely, it is routine for people with FS to be diagnosed with epilepsy for many years before the diagnosis is revised,^21^ and in a clinical database, that earlier diagnosis will remain in place. Preceding mental health diagnoses will likely bias clinicians toward diagnosing FS, whether rightly^22^ or wrongly,^23^ and the noticeable levels of pathology in our group defined by having no health problems on examination shows at scale the lack of certainty of medical assessments in general. Although diagnosed prior to the diagnosis of FS, it is possible that our “baseline” comorbidities are nonetheless mediators of the mortality that follows, and therefore that correcting for them may produce an overadjustment bias. We note, however, that the unmatched results support the same conclusions.

Our results nevertheless present an alarming risk of premature death in people with FS, which although still only half of that in people with ES, is still more than twice that in NS and is not attributable to the many comorbidities found in FS. Although the causes of death are not explained by these data, and would benefit from future elucidation, they should provide further motivation for clinicians and services to improve engagement and therapies for people with FS.

## AUTHOR CONTRIBUTIONS

Richard A. Kanaan designed the study, conducted the analyses, and acts as guarantor. All authors wrote the manuscript.

## CONFLICT OF INTEREST STATEMENT

R.A.K. does medical expert reporting in personal injury and clinical negligence cases, including in cases of functional neurological disorder (FND); receives royalties from Guildford Press for a chapter on FND and feigning; has received grant funding, including for studies related to FND, from the UK Medical Research Council and the Wellcome Trust; and receives grant funding from the Australian National Health and Medical Research Council, Medical Research Future Fund, Weary Dunlop Foundation, and Victorian Government. R.B. is supported by the Slovenian Research and Innovation Agency as a member of the research program Medical Physics (P1‐0389). T.P. does medical expert reporting in personal injury and clinical negligence cases, including in cases of FND. He has received consultancy fees from Arialys Therapeutics. M.R. has received research funding from the NIHR and Epilepsy Research UK. He receives a salary as Editor in Chief for *Seizure: European Journal of Epilepsy*. He has received honoraria from Angelini Pharma, UCB Pharma, Jazz Pharma, LivaNova, and Precisis. He has received royalties from Oxford University Press and Jessica Kingsley Publishers. S.P. is supported by Advanced Clinician Scientist Programme UMEA2 (01EO2104) from the German Federal Ministry of Research, Technology, and Space. He receives royalties from Springer Nature for a book on functional neurological disorders. We confirm that we have read the Journal's position on issues involved in ethical publication and affirm that this report is consistent with those guidelines.

## Supporting information


Table S1.

## Data Availability

The data that support the findings of this study are available from TriNetX. Restrictions apply to the availability of these data, which were used under license for this study. Data are available from https://trinetx.com/solutions/live‐platform/ with the permission of TriNetX.
