# Supplementary material for: Mortality in functional seizures: Evidence from a large electronic health records dataset
Source: Epilepsia. 2026 Mar 31;67(7):3709–18. doi: 10.1002/epi.70230 (PMC13360918; doi:10.1002/epi.70230)
Supplement: Supplementary file 1 — Table S1. [file EPI-67-3709-s001.docx]

Supplementary Table 1

| **Demographics and Clinical Conditions present in those with two or more diagnoses of functional seizures (N = 11,831) and all those with functional seizures (N = 32,492)** | | | | | | | | | | | | | | | |
| --- | --- | --- | --- | --- | --- | --- | --- | --- | --- | --- | --- | --- | --- | --- | --- |
|  | **Demographics** | | | | | | | | | | | | | | |
|  |  | Cohort | |  | | Mean ± SD | | Patients | | | % of Cohort | | P-Value | Std diff. | |
|  |  | F2 FS | AI | Age at Index | | 31.9 +/- 17.4 34.7 +/- 19.5 | | 11,814 32,362 | | | 100% 100% | | <0.001 | 0.153 | |
|  |  | F2 FS | 2106-3 | White | |  | | 7,798 18,843 | | | 66.0% 58.2% | | <0.001 | 0.161 | |
|  |  | F2 FS | 2054-5 | Black or African American | |  | | 1,842 4,748 | | | 15.6% 14.7% | | 0.016 | 0.026 | |
|  |  | F2 FS | M | Male | |  | | 2,497 8,064 | | | 21.1% 24.9% | | <0.001 | 0.090 | |
|  |  | F2 FS | 2028-9 | Asian | |  | | 166 445 | | | 1.4% 1.4% | | 0.811 | 0.003 | |
|  | **Diagnosis** | | | | | | | | | | | | | | |
|  |  | Cohort | | |  | |  | | Patients | % of Cohort | | P-Value | | | Std diff. |
|  |  | F2 FS | F30-F39 | | Mood [affective] disorders | |  | | 3,658 8,198 | 31.0% 25.3% | | <0.001 | | | 0.125 |
|  |  | F2 FS | F10-F19 | | Mental and behavioral disorders due to psychoactive substance use | |  | | 1,582 4,196 | 13.4% 13.0% | | 0.241 | | | 0.013 |
|  |  | F2 FS | F50-F59 | | Behavioral syndromes associated with physiological disturbances and physical factors | |  | | 686 1,407 | 5.8% 4.3% | | <0.001 | | | 0.066 |
|  |  | F2 FS | F90-F98 | | Behavioral and emotional disorders with onset usually occurring in childhood and adolescence | |  | | 1,184 2,412 | 10.0% 7.5% | | <0.001 | | | 0.091 |
|  |  | F2 FS | F80-F89 | | Pervasive and specific developmental disorders | |  | | 441 950 | 3.7% 2.9% | | <0.001 | | | 0.044 |
|  |  | F2 FS | F20-F29 | | Schizophrenia, schizotypal, delusional, and other non-mood psychotic disorders | |  | | 384 1,049 | 3.3% 3.2% | | 0.963 | | | 0.001 |
|  |  | F2 FS | F60-F69 | | Disorders of adult personality and behavior | |  | | 562 1,221 | 4.8% 3.8% | | <0.001 | | | 0.049 |
|  |  | F2 FS | F70-F79 | | Intellectual Disabilities | |  | | 105 232 | 0.9% 0.7% | | 0.066 | | | 0.019 |
|  |  | F2 FS | F41 | | Other anxiety disorders | |  | | 3,847 8,437 | 32.6% 26.1% | | <0.001 | | | 0.143 |
|  |  | F2 FS | F43 | | Reaction to severe stress, and adjustment disorders | |  | | 1,618 3,494 | 13.7% 10.8% | | <0.001 | | | 0.089 |
|  |  | F2 FS | F42 | | Obsessive-compulsive disorder | |  | | 208 412 | 1.8% 1.3% | | <0.001 | | | 0.040 |
|  |  | F2 FS | F45 | | Somatoform disorders | |  | | 253 579 | 2.1% 1.8% | | 0.016 | | | 0.025 |
|  |  | F2 FS | J00-J99 | | Diseases of the respiratory system | |  | | 3,570 8,583 | 30.2% 26.5% | | <0.001 | | | 0.082 |
|  |  | F2 FS | M00-M99 | | Diseases of the musculoskeletal system and connective tissue | |  | | 4,563 11,024 | 38.6% 34.1% | | <0.001 | | | 0.095 |
|  |  | F2 FS | E00-E89 | | Endocrine, nutritional and metabolic diseases | |  | | 3,780 9,177 | 32.0% 28.4% | | <0.001 | | | 0.079 |
|  |  | F2 FS | I00-I99 | | Diseases of the circulatory system | |  | | 3,048 7,873 | 25.8% 24.3% | | 0.002 | | | 0.034 |
|  |  | F2 FS | K00-K95 | | Diseases of the digestive system | |  | | 3,553 8,716 | 30.1% 26.9% | | <0.001 | | | 0.070 |
|  |  | F2 FS | N00-N99 | | Diseases of the genitourinary system | |  | | 3,006 7,248 | 25.4% 22.4% | | <0.001 | | | 0.071 |
|  |  | F2 FS | G00-G99 | | Diseases of the nervous system | |  | | 4,938 11,915 | 41.8% 36.8% | | <0.001 | | | 0.102 |
|  |  | F2 FS | A00-B99 | | Certain infectious and parasitic diseases | |  | | 2,095 5,070 | 17.7% 15.7% | | <0.001 | | | 0.055 |
|  |  | F2 FS | L00-L99 | | Diseases of the skin and subcutaneous tissue | |  | | 1,996 4,790 | 16.9% 14.8% | | <0.001 | | | 0.057 |
|  |  | F2 FS | H00-H59 | | Diseases of the eye and adnexa | |  | | 1,561 3,732 | 13.2% 11.5% | | <0.001 | | | 0.051 |
|  |  | F2 FS | D50-D89 | | Diseases of the blood and blood-forming organs and certain disorders involving the immune mechanism | |  | | 1,502 3,617 | 12.7% 11.2% | | <0.001 | | | 0.047 |
|  |  | F2 FS | H60-H95 | | Diseases of the ear and mastoid process | |  | | 1,169 2,697 | 9.9% 8.3% | | <0.001 | | | 0.054 |
|  |  | F2 FS | O00-O9A | | Pregnancy, childbirth and the puerperium | |  | | 454 1,094 | 3.8% 3.4% | | 0.019 | | | 0.025 |

F2: Two diagnoses of functional seizures; FS: One or more diagnoses of functional seizures; SD: standard deviation; Std Diff: standardised difference.
